# Identity of Low-Molecular-Weight Species Formed in End-To-End Cyclization Reactions Performed in THF

**DOI:** 10.3390/polym10080844

**Published:** 2018-07-31

**Authors:** Ching W. Pan, Katherine Xia, Samantha A. Parker, Eric S. Tillman

**Affiliations:** Department of Chemistry and Biochemistry, Santa Clara University, 500 El Camino Real, Santa Clara, CA 95053, USA; cpan@scu.edu (C.W.P.); kxia@scu.edu (K.X.); saparker@scu.edu (S.A.P.)

**Keywords:** cyclic polymers, click chemistry, atom transfer radical coupling

## Abstract

Cyclic polymers were produced by end-to-end coupling of telechelic linear polymers under dilute conditions in THF, using intramolecular atom transfer radical coupling or click chemistry. In addition to the expected shift to longer elution times on gel permeation chromatography (GPC) indicative of the formation of cyclic product, lower molecular weight species were consistently observed upon analysis of the unpurified cyclization reaction mixture. By systematically removing or altering single reaction components in the highly dilute cyclization reaction, it was found that THF itself was responsible for the low-molecular-weight material, forming oligomeric chains of poly(THF) regardless of the other reaction components. When the reactions were performed at higher concentrations and for shorter time intervals, conducive to intermolecular chain-end-joining reactions, the low-molecular-weight peaks were absent. Isolation of the material and analysis by ^1^H NMR confirmed that poly(THF) was being formed in the highly dilute conditions required for cyclization by end-to-end coupling. When a series of mock cyclization reactions were performed with molar ratios of the reactants held constant, but concentrations changed, it was found that lower concentrations of reactants led to higher amounts of poly(THF) side product.

## 1. Introduction

**Scheme 1:**
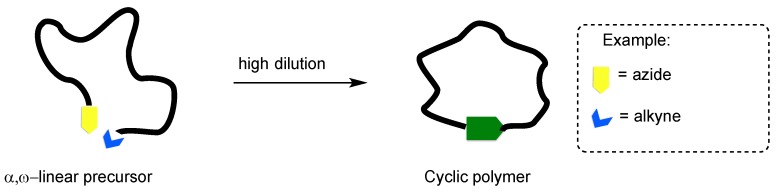
Synthesis of cyclic polymers by end-to-end coupling of a difunctional polymer precursor. The yellow and blue chain ends may represent any compatibly reactive groups, with azide and alkyne listed as an example.

Methods such as ring opening metathesis [1] and polyhomologation [2] can lead to macrocycles, yet perhaps the most common technique involves the chain-end closure of telechelic linear polymer precursors. This much-used sequence will often follow the same general plan: synthesize α,ω-telechelic polymers with reactive chain ends, and coerce an intramolecular coupling reaction by appropriate choice of chain ends and dilution (Scheme 1). This approach is conceptually simple, with the overall process involving end-group transformations of one or both of the chain ends to prepare the linear precursors capable of cyclization. Examples of reactive chain ends for cyclization include, but are not limited to: (i) alkynes and azides for “click” reactions [3,4,5]; (ii) dienes and dienophiles for Diels–Alder chemistry [6]; (iii) alkenes and thiols for thiol-ene reactions [7]; (iv) anthracenes for photodimerization reactions [8]; (v) carbanions for reactions with external electrophiles (by consecutive S_N_2 reactions between a polymer anion and bifunctional electrophile) [9,10]; and (vi) C–Br bonds capable of activation in a radical transfer coupling sequence [11,12].

A common solvent for both polymerizations, leading to linear precursors and post-polymerization transformation reactions, is tetrahydrofuran (THF). This solvent is compatible with all the cyclization reactions listed above, while also being a compatible polymerization medium for anionic techniques [13,14] and reversible activation/deactivation radical polymerization (RDRP) methods such as atom transfer radical polymerization (ATRP) [15,16] and nitroxide-mediated polymerization (NMP) [17]. The success of cyclization reactions is almost always evaluated, at least in part, using gel permeation chromatography (GPC), with the shift to longer elution times indicative of the topological transformation from linear to cyclic [18,19]. Further analysis, such as via MALDI-TOF [20,21,22], is often used to verify the cyclic structure and validate the proposed mechanistic pathway leading to its creation.

In this submission, the outcomes of intramolecular, chain-end azide–alkyne “click” reactions and atom transfer radical coupling (ATRC) reactions, both leading to the formation of macrocyclic polystyrene (PS), were analyzed. All cyclization reactions were performed under highly dilute conditions in THF, and the raw cyclization reaction mixtures, concentrated by rotary evaporation, were analyzed by GPC. In addition to the expected signals attributed to cyclic material and higher-molecular-weight species formed by competing intermolecular chain-end coupling, signals at lower molecular weights (~1100 Da) were consistently observed. This had previously been seen by our group in trials using ATRC-type ring closing reactions, but was never investigated. By systematically altering and removing reaction mixture contents, the identity of the low-molecular-weight species was determined and is reported here. 

## 2. Materials and Methods

### 2.1. Materials

Copper(I) bromide (CuBr, 98%, Aldrich, St. Louis, MO, USA) and copper metal (Cu^0^, fine powder, Baker & Adamson, Easton, PA, USA) were used as received. Tetrahydrofuran (THF, inhibitor free ≥99.9%, Aldrich, St. Louis, MO, USA) was obtained from the Aldrich Pure-Pac^TM^ System. Styrene (S, ≥99%, Aldrich) was purified through prepacked column inhibitor removers (Aldrich). 2-Azidoethyl-2-bromoisobutyrate (Aldrich) and 1,6-heptadiyne (Aldrich) were used as received. The following were used as received and stored in the refrigerator: 1-bromoethylbenzene (BEB, 97%, Aldrich), α,α-dibromotoluene (97%, Aldrich) and *N*,*N*,*N*′,*N*″,*N*″-pentamethyldiethylenetriamine (PMDETA, 99%, Aldrich). The *N*-*tert*-butyl-α-phenylnitrone (tBuPN, Aldrich) and 2-methyl-2-nitrosopropane (MNP, Aldrich) were used as received and stored in the freezer.

### 2.2. Procedure for the Synthesis of Monobrominated Polystyrene Using ATRP

Styrene (4.0 mL, 34.9 mmol), CuBr (100 mg, 0.698 mmol), and (1-bromoethyl)benzene (BEB, 95 μL, 0.698 mmol) were added into a custom-made Schlenk flask, capped with a rubber septum, that was attached to the Schlenk line. The contents of the flask were exposed to three freeze−pump−thawing cycles before being placed on a heat plate with custom-fit block heaters and equipped with a digital contact thermoregulator (Chemglass CG-1994-V015) set to 80 °C and left to stir for 5 min. PMDETA (146 μL, 0.698 mmol) was then added to the reaction flask via syringe to begin polymerization. Dibrominated polystyrene was synthesized using the same material as listed above except using α,α-dibromotoluene (115 μL, 0.698 mmol) as the initiator instead of BEB.

### 2.3. Typical Procedure for the ATRC of PSBr

A representative ATRC reaction was performed as follows: a molar ratio of [PsBr]:[CuBr]:[Cu^0^]:[PMDETA] = 1:5:5:10, monobrominated polystyrene (PSBr, 195 mg, 0.075 mmol, 2600 g/mol), CuBr (53.8 mg, 0.375 mmol), Cu^0^ (16 mg, 0.375 mmol), and THF (5 mL) were added into a custom-made Schlenk line round-bottom flask. Concentrations of PSBr were typically kept at 15 mM for intermolecular reactions. The reaction flask, sealed with a rubber septum, was attached to the Schlenk line. The flask was exposed to three freeze−pump−thawing cycles before being placed onto a heat plate set to 60 °C. After 5 min of heating and stirring, PMDETA (157 μL, 0.75 mmol) was added into the reaction chamber via syringe. The coupling reaction was stopped after 60 min by exposing the content of the flask to the atmosphere and placing the flask in an ice bath. The reaction mixture was filtered into a GPC vial, diluted with THF, and analyzed. A radical trap-assisted ATRC (RTA-ATRC) reaction was performed using the same material listed above with the addition of *N*-*tert*-butyl-α-phenylnitrone (tBuPN, 13 mg, 0.075 mmol), and then following the same procedure described.

### 2.4. Typical Procedure for the End-To-End Coupling Condition, Using the Highly Dilute RTA-ATRC of PSBr as an Example

A representative highly dilute RTA-ATRC reaction was performed as follows: a molar ratio of [BrPsBr]:[CuBr]:[Cu^0^]:[PMDETA] = 1:20:20:40, dibrominated polystyrene (BrPSBr, 13 mg, 0.005 mmol, 2700 g/mol), CuBr (214.3 mg, 0.1 mmol), Cu^0^ (6.32, 0.1 mmol), 2-methyl-2-nitrosopropane dimer (MNP, 157 mM MNP-dimer solution in THF, 160 μL, 0.025 mmol), and THF (50 mL) were added into a custom-made Schlenk line round-bottom flask. Concentrations of BrPSBr were consistently kept at 10^−4^ mM. The reaction flask, sealed with a rubber septum, was attached to the Schlenk line. The flask was exposed to three freeze−pump−thawing cycles before being placed onto a heat plate set to 60 °C. After 5 min of heating and stirring, PMDETA (42 μL, 0.2 mmol) was added into the reaction chamber via syringe. The RTA-ATRC reaction was stopped after 24 h by exposing the content of the flask to the atmosphere and placing the flask in an ice bath. The reaction mixture was filtered into a GPC vial, diluted with THF, and analyzed. Analogous end-to-end coupling reactions were conducted using azide–alkyne “click” chemistry at similar concentrations.

### 2.5. Procedure of Synthesis of α,ω-Diazido PS

N_3_–SBr was synthesized using established ATRP methods and 2-azidoethyl-2-bromoisobutyrate as the initiator. N_3_–PS–N_3_ was synthesized subsequently using established RTA-ATRC methods with N_3_–PSBr and MNP as the radical trap.

### 2.6. Characterization

Raw reaction products were placed on a rotary evaporator (Buchi Rotavapor R-114, New Castle, DE, USA) to concentrate the crude products for GPC analysis. GPC analysis was done on a system comprised of a Shimadzu CBM-20A communications module, a Shimadzu DGU-20A degassing unit, a Shimadzu SIL-20A auto sampler, a Shimadzu LC-20AD pump, and a Wyatt T-rEX RI detector (Santa Barbara, CA, USA). The instrument was interfaced with a PC and was operated using PSS WinGPC Unichrom software (Mainz, Germany). Separations were performed using a PSS SDV analytical 1000-angstrom and a 100,000-angstrom column in sequence, housed inside a Shimadzu CTO-2A column oven set (Kyoto, Japan) to 40 °C. THF was used as an eluent at an optimized flow rate of 1.00 mL/min, and a 10-point calibration was performed using Agilent EasiCal PS standards. The data analysis was performed using the PSS WinGPC software.

## 3. Results

**Scheme 2:**
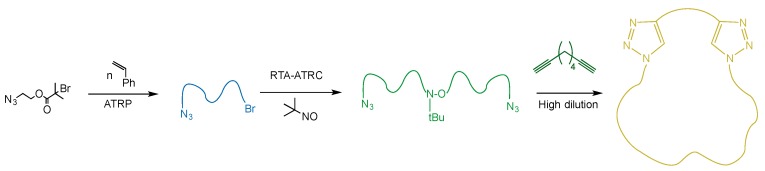
Preparation of cyclic PS using consecutive “click” reactions. Characteristics of the precursor and products are shown in Figure 1.

**Figure 1:**
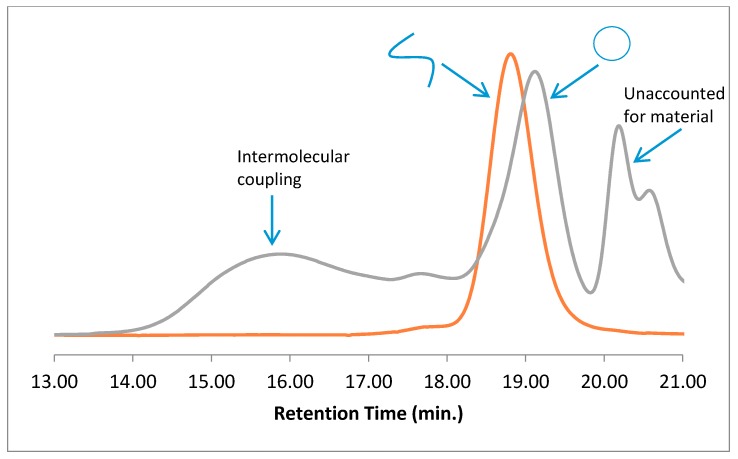
GPC traces of N_3_–PS–N_3_ precursor (orange) and unpurified product of intramolecular “click” cyclization reaction (gray). “Click” reaction was conducted at 60 °C and [N_3_–PS–N_3_] of 0.1 mM. Precursor characteristics: *M*_n_: 3900 Da, *M*_p_: 4150 Da, Đ: 1.11. Cyclic product characteristics: *M*_p_: 3300 Da, <G> = 0.80.

The results presented in this paper are focused not on assessing the yields or purity of the cyclic polymers, as the methods used have already been reported and proven capable of producing macrocycles [5,12]. Rather, the focus is on elucidating the identify of a low-molecular-weight species whose presence cannot be accounted for by the chemistry used to close the polymer chains into rings. To this end, linear polystyrene with diazide termini was prepared by a combination of ATRP and radical trap-assisted ARTC, and subjected to consecutive “click” reactions with a diyne, with the second reaction being intramolecular. The synthetic route is summarized in Scheme 2 and detailed in Materials and Methods, and the GPC traces of both the precursor and cyclization reaction mixture are presented in Figure 1 along with the characteristics of the polymers. Typically of intramolecular chain-end-joining reactions, a shift to longer elution times indicates the presence of cyclic material [20], while the presence of material with a broad range of molecular weights shows that intermolecular coupling and “step” polymerization is competing with cyclization, although some cyclization occurring after step-growth cannot be ruled out [23,24]. The low-molecular-weight species that elute past 20 min and correspond to apparent molecular weights of near 1100 Da are too high to be residual monomer or ligand/catalyst complexes, yet cannot be explained by any expected “click” chemistry.

**Scheme 3:**
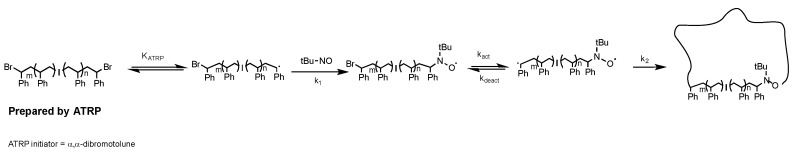
Preparation of cyclic PS using intramolecular RTA-ATRC. Characteristics of the precursor and products are shown in Figure 2.

**Figure 3:**
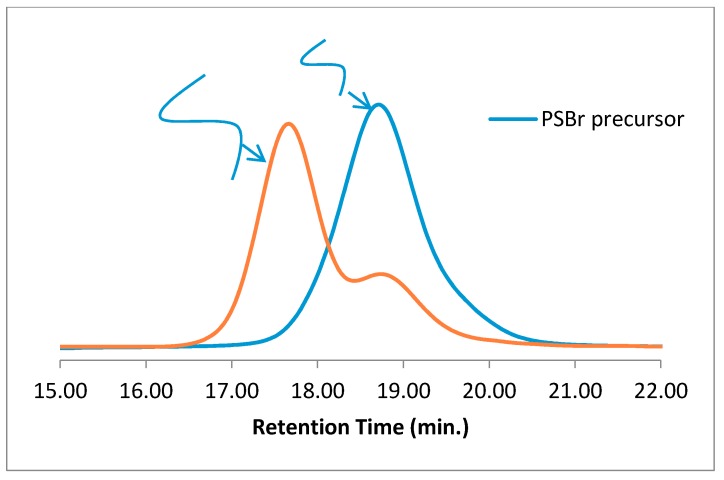
GPC traces of PSBr precursor (blue) and unpurified product of dimerization reaction (orange). Note that this is not a cyclization reaction, but a dimerization reaction performed at higher concentrations. RTA-ATRC reaction was conducted at 60 °C and [PSBr] of 15 mM. Precursor characteristics: *M*_n_: 3800 Da, *M*_p_: 4600 Da, Đ: 1.2. Coupled product characteristics: *M*_n_: 6400 Da, *M*_p_: 9600 Da, Đ: 1.31.

When cyclic polymers were prepared by a different route, using intramolecular RTA-ATRC (Scheme 3), similar, unaccounted-for material was observed: the expected signals attributed to cyclic material and “step” products, but also the low-molecular-weight material at nearly the exact same elution volume. The cyclization reaction mixture contained many of the same reactants as the “click” cyclization reaction, and this led us to further consider if some combination of ligand/catalyst and polymer was leading to these low-molecular-weight impurities. Interestingly, when an intermolecular chain-end-joining reaction of RTA-ATRC was carried out with monobrominated PS under much higher reactant concentrations, this low-molecular-weight material was not observed in the unpurified reaction mixture. This is consistent with our previous studies that used ATRC-type reactions to prepare polymeric dimers [12]. The GPC traces of the precursor and reaction mixture are shown in Figure 3, with signals corresponding to only the coupled product and residual precursor found in the GPC traces of the product.

**Figure 4:**
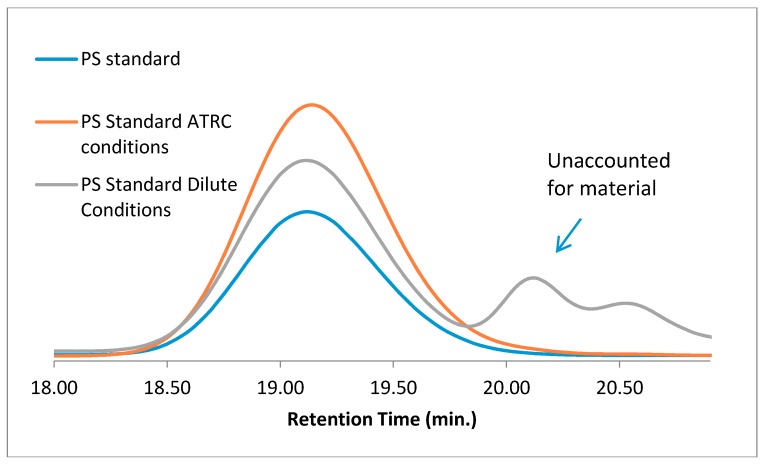
GPC traces of unfunctionalized PS (Blue; purchased from Aldrich, St. Louis, MO, USA) and unpurified product of mock “dimerization reaction” (orange) and mock, high-dilution “cyclization reaction”. Note that these simply mimic cyclization or ATRC reactions, using a nonreactive polymer incapable of chain-end-joining reactions. ATRC reaction conditions: 60 °C and [PS standard] = 15 mM. Dilute reaction conditions of 60 °C and [PS standard] = 0.1 mM. Polystyrene standard characteristics: *M*_n_: 3100 Da; *M*_p_: 3300 Da.

To remove the possibility of having reactive polymer chain ends undergoing some sort of depolymerization under highly dilute conditions, PS analytical standards were used as a “linear precursor” and subjected to cyclization reaction conditions and more concentrated dimerization conditions. As shown in Figure 4, under more concentrated ATRC-type conditions, the only signal observed was that of the original PS standard, while under highly dilute conditions mimicking cyclization conditions, the unaccounted for, low-molecular-weight material was also observed. This rules out the reactive functional groups on the PS chain ends being responsible for creating low-molecular-weight materials.

**Figure 5:**
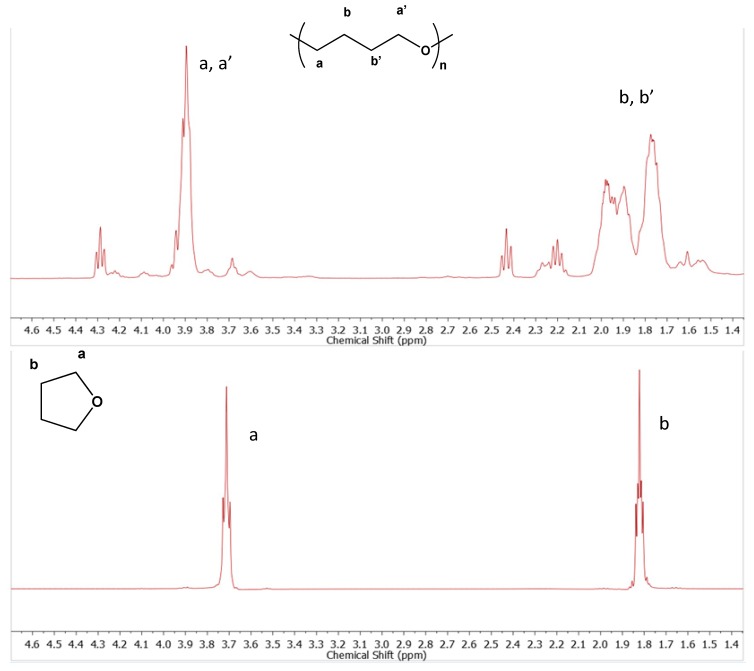
^1^H NMR spectra of THF (bottom) and poly(THF) formed with only 15 mM of CuBr as the sole reactant, and subjected to cyclization conditions of 60 °C while 24 h stirring.

Removing the polymer, ligand, and radical trap and only having CuBr in THF at the concentration and temperature used for cyclization reactions, perhaps surprisingly, resulted in identical signals to those observed in the earlier cyclization reactions. It should be noted that, as a test, when the same components were prepared and immediately analyzed by GPC, no such signals were observed; only after the reaction mixture was stirred at cyclization temperature (60 °C) for the allotted time did these signals appear on GPC. This low-molecular-weight material was isolated as a sticky gel by rotary evaporation of the reaction mixture and analyzed by ^1^H NMR after removal of residual copper. At this point, it was clear that the material must arise from THF itself, and a ^1^H NMR spectrum of THF was compared to that of the isolated low-molecular-weight material. As shown in Figure 5, the material is consistent with low-molecular-weight poly(THF) [25] and, by process of elimination, must be poly(THF). Smaller, sharper signals around 4.4 ppm and between 2.2–2.5 ppm may be due to smaller, oligomeric chains and/or end groups. The GPC data shows some variety in the length and apparent size of these side products, and future work needs to be done for a more thorough structural assignment and mechanistic explanation. Interestingly, this side product is only formed under highly dilute conditions, and while the exact mechanism is not clear, it could result from trace amounts of a protic source or simply complexation with CuBr and another THF molecule acting as a nucleophile.

**Figure 6:**
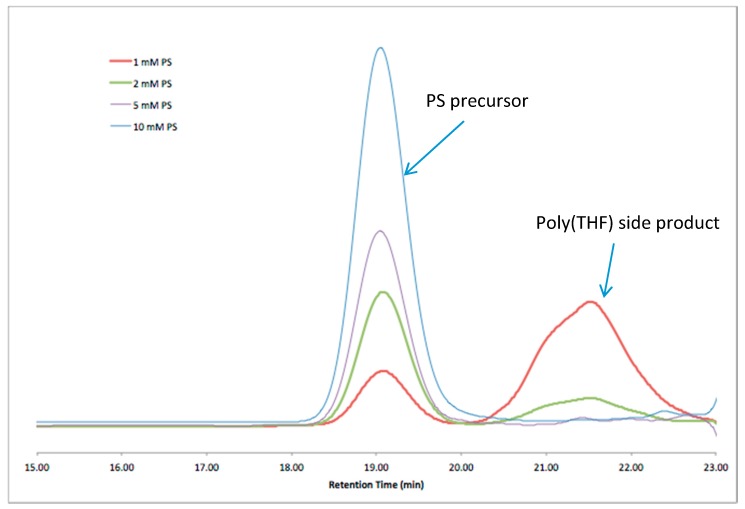
GPC traces of unfunctionalized PS (purchased from Agilent, eluting between 18.5 and 20.0 min) and the results of several mock cyclization reactions at varying concentrations of reactants ranging from 1 mM PS to 10 mM PS. In all cases, [PS]:[CuBr]:[Cu]:[PMDETA] = 1:5:5:10.

A series of mock cyclizations were performed, using various concentrations of PS standards (*M*_n_ = 2970 Da) and reactants to mimic the polymer precursor and commonly used molar ratios of copper bromide, copper, and PMDETA ligand. The goal of these series of reactions was to relate reactant concentrations to the evolution and the extent to which the unwanted poly(THF) was observed. As shown in Figure 6, greater relative amounts of poly(THF) side products, compared to PS, were found at lower concentrations of reactants overall. Note that these reaction protocols differed when compared to the standard intermolecular ATRC-type conditions (Figure 4) in that they were allowed to proceed for 24 h to match a time length applicable to cyclization conditions. The results of Figure 6 are consistent with the side product being formed in highly dilute conditions, but its production was absent or minimized when reactant concentrations were increased.

**Figure 7:**
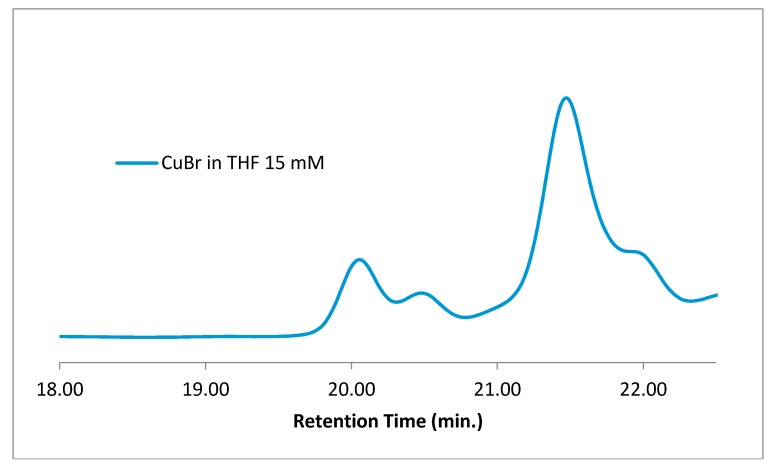
GPC trace of results for CuBr and THF, with all other reactants absent, stirred at 60 °C for 24 h with [CuBr] at 15 mM. The reaction mixture was concentrated under reduced pressure and analyzed by GPC. Note: if the mixture was analyzed prior to heating, no poly(THF) was observed.

Lastly, to demonstrate that the low-molecular-weight species is formed from THF and is not somehow related to the other species in the mock cyclization reactions, removing the polymer, ligand, and radical trap and only having CuBr in THF, surprisingly, resulted in the identical signals being observed (Figure 7). It should be noted that, as a test, when the same components were prepared and immediately analyzed by GPC, no such signals were observed; only after the reaction mixture was stirred at cyclization temperature (60 °C) for the allotted time did these signals appear on GPC.

## 4. Conclusions

When performed in THF under highly dilute conditions, low-molecular-weight material was observed in the raw, concentrated cyclization reaction mixture for both azide–alkyne “click” and ATRC-type reaction conditions, regardless of the structure or even presence of the linear polymer precursor. This low-molecular-weight material was identified as poly(THF), and can be minimized or avoided by adjusting the concentrations of the reactants. This material can be easily removed by precipitation of the cyclic polymer, and does not appear to affect the success of the reaction in any way. Its presence in the GPC traces of the unpurified cyclization reaction components had been previously puzzling because it could not be accounted for by the expected reaction pathways of the linear polymeric reactants and known mechanisms of cyclization.

## Figures and Tables

**Figure 2:**
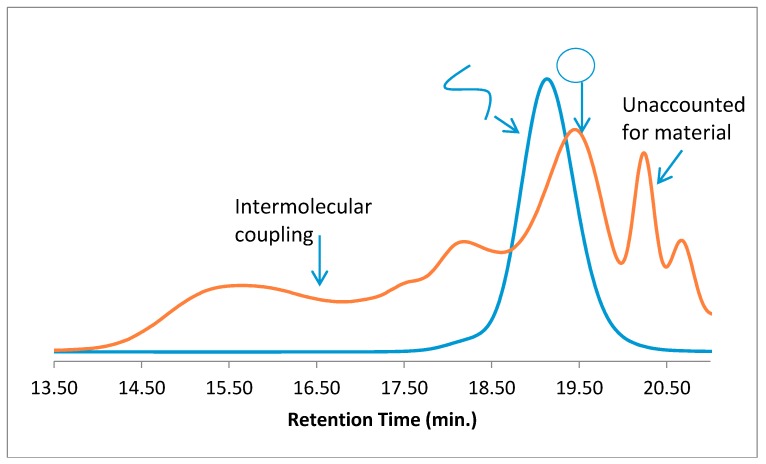
GPC traces of BrPSBr precursor (orange) and unpurified product of intramolecular RTA-ATRC reaction (orange). RTA-ATRC reaction conducted at 60 °C and [BrPSBr] of 0.1 mM. Precursor characteristics: *M*_n_: 3100 Da, *M*_p_: 3250 Da, Đ: 1.09. Cyclic product characteristics: *M*_p_: 2600 Da, <G> = 0.8.
